# Transcondylar fossa approach for the large, high-flow, and diffuse arteriovenous malformation of the posterior fossa

**DOI:** 10.3171/2020.10.FOCVID2045

**Published:** 2021-01-01

**Authors:** Koichi Torihashi, Takafumi Ogura, Mitsutoshi Kadowaki, Makoto Sakamoto, Masamichi Kurosaki

**Affiliations:** Division of Neurosurgery, Department of Brain and Neurosciences, Faculty of Medicine, Tottori University, Tottori, Japan

**Keywords:** arteriovenous malformation, posterior fossa, high grade, cerebellopontine angle, video

## Abstract

Posterior fossa high-grade arteriovenous malformations (AVMs) are challenging diseases. This video presents the treatment of a patient with a diffuse, high-flow AVM of the posterior fossa on the tonsil and cerebellopontine angle (4 cm in diameter) and deep draining veins. The patient had an intraventricular and cerebellar hemorrhage. After conservative treatment, total resection of the AVM was performed with embolization and surgery. The authors resected the nidus after the endovascular embolization, on the same day. The postoperative course was uneventful, and the patient was discharged with almost full recovery.

The video can be found here: https://youtu.be/logCCn3uKUc

## Transcript

Posterior fossa high-grade arteriovenous malformations (AVMs) are challenging diseases.^1,2^ Therefore, the treatment for posterior fossa AVM is recommended only for hemorrhage cases.^3^ However, most posterior fossa AVMs are associated with an increased hemorrhage rate.^4^

This video presents the treatment of a patient with a posterior fossa AVM on the tonsil and cerebellopontine angle, 4 cm in diameter (diffuse, high flow), and deep draining veins. This AVM was Spetzler-Martin grade III and located on the tonsil and cerebellopontine angle.^5,6^

The patient had intraventricular and cerebellar hemorrhage. After conservative treatment, total resection of the AVM was performed with embolization and surgical resection. We resected the nidus on the same day after the endovascular embolization. Postoperative course was uneventful, and he was discharged almost fully recovered.

**0:21 Introduction.** This video describes the surgical treatment of a large, high-flow, and diffuse AVM of the posterior fossa.

**0:31 Clinical History.** The patient was a 37-year-old male. He presented with a loss of consciousness, headache, and dizziness. Neurological examination revealed ataxia. He was found to have intraventricular and cerebellar hemorrhage. After conservative treatment and observation, he was transferred to our institution.

**0:53 Preoperative Imaging.** Computed tomography (CT) demonstrated left cerebellar and intraventricular hemorrhage. Magnetic resonance imaging (MRI) demonstrated the AVM at the tonsil and cerebellopontine angle. The nidus measured 4 cm in diameter.

Angiography of the left vertebral artery (VA) demonstrated a large, high-flow, and diffuse AVM of the posterior fossa. 3D digital subtraction angiography demonstrated that the nidus was fed from the left anterior inferior cerebellar artery (AICA), left posterior inferior cerebellar artery (PICA), and several feeders around the occipital sinus. The deep draining veins enter the bilateral superior petrosal sinus (SPS), and the superficial draining veins enter the occipital sinus (Supplemental Fig. 1).

In terms of the Spetzler-Martin grade, this AVM was grade III and located on the tonsil and cerebellopontine angle.

**1:48 Feeding Arteries and Draining Veins of the AVM.** There are three major groups of arterial feeding arteries supplying the AVM: one group from the AICA, the second group from the PICA, and many feeders around the occipital sinus. All feeders are intradural feeding arteries. The AVM has three draining veins. Deep draining veins align to the bilateral SPS, and the superficial draining vein terminates to the occipital sinus.

**2:16 Operative Procedure.** Preoperative embolization is useful to reduce the flow of the AVM.^7^ In this procedure, the AICA and PICA were embolized using endovascular treatment with Onyx. It is hard to identify the AICA on the operative field because it is derived from the ventral side of the nidus. After obliteration of the AICA and PICA, the AVM was operated on in the operating room on the same day.

At first, the main feeder of the PICA was identified and the feeders around the superior surface of the nidus and the occipital sinus were dissected. Then, from the suboccipital surface to the petrosal surface was dissected. The drainer to the left SPS was identified. The main feeders of the AICA and PICA were separated. After all feeders were separated, the drainers were dissected and separated, and thereafter the nidus was removed.

**3:13 Endovascular Treatment.** The patient underwent multiple embolization procedures in which the AICA and PICA were embolized before surgical resection. Onyx was injected through the microcatheter. The angiogram before surgery revealed continued slowing and thrombosis of the nidus.

3:38 Positioning, Skin Incision, and Craniotomy. The patient was put in the park-bench position. The head was slightly turned with the contralateral side downward. A hockey-stick incision was made. The craniotomy was extended to the condylar fossa. The foramen magnum was opened and C1 laminectomy was carried out.

**3:57 Operative Video.** The resection of the AVM was performed by the left transcondylar fossa approach on the same day of the endovascular treatment 35 days after the onset. The posterior fossa AVM resection is recommended 4–6 weeks after the onset due to mass effect.^8^

**4:00** Pial and parenchymal dissection was carefully undertaken around the superior surface of the nidus.

**4:12** The main feeder of the PICA was identified.

**4:18** The feeders around the main draining vein to the occipital sinus and the superior surface of the nidus were individually dissected, coagulated, and separated. The arachnoid and pial dissection was carefully performed.

**4:46** The deepest part of the nidus was the hematoma cavity. We can see the hematoma cavity. The bleeding artery was coagulated and Surgicel cotton balls were used for hemostasis.

**5:01** The medial side of the main draining vein was dissected. A Weck clip was used when necessary to occlude and separate the feeders.

**5:15** The area from the occipital surface to the petrosal surface was also dissected. We can see the vertebral artery. The arachnoid around the lower cranial nerves was dissected, and the draining vein going to the SPS was exposed. We used the operative view from the transcondylar fossa to identify the vertebral artery and dissect the arachnoid around the lower cranial nerves.

**5:35** After separating the feeding arteries, the PICA was exposed and clipped. ICG angiography was used to ascertain blood flow. Having checked the flow of the AVM, the PICA was clipped, coagulated, and separated.

**5:58** Feeding arteries were separated on the ventral side of the nidus. We can see the AICA. The AICA was embolized by cast Onyx. The intravascular Onyx was then dissected.

**6:17** The draining vein going to SPS was separated, and the last feeder was separated.

**6:27** After the complete occlusion of all arteries, the nidus was movable. The draining vein going to the occipital sinus was separated.

**6:39** The AVM was totally resected.

**6:50 Postoperative Course.** The postoperative course was uneventful. The patient experienced no other neurological deficit. He was discharged 3 weeks after surgery.

**6:58 Postoperative Imaging.** Postoperative MRI demonstrated a minor cerebellar contusion but no cerebral ischemia. Postoperative angiography demonstrated total removal of the AVM and preservation of the passing artery.

**7:17 Patient Walking on Postoperative Day 10.** The patient was able to move independently without any assistance. He could walk smoothly and returned to normal life.

## Author Contributions

Primary surgeon: Torihashi. Assistant surgeon: Ogura, Kadowaki, Sakamoto, Kurosaki. Editing and drafting the video and abstract: Torihashi. Reviewed submitted version of the work: Torihashi. Approved the final version of the work on behalf of all authors: Torihashi. Supervision: Kurosaki. Endovascular surgeon: Sakamoto, Kadowaki.

## Supplemental Information

Supplemental Fig. 1
