# Supplementary material for: Transcondylar fossa approach for the large, high-flow, and diffuse arteriovenous malformation of the posterior fossa
Source: Neurosurg Focus Video. 2021 Jan 1;4(1):V5. doi: 10.3171/2020.10.FOCVID2045 (PMC9542486; doi:10.3171/2020.10.FOCVID2045)
Supplement: Supplemental Fig. 1 [file SupplementalFig1_FOCVID20-45.pdf]

NEUROSURGICAL
FOCUS
VIDEO

ONLINE ONLY

Supplemental material

Transcondylar fossa approach for the large, high-flow, and diffuse arteriovenous malformation of the posterior fossa

Torihashi et al.

https://thejns.org/doi/abs/10.3171/2020.10.FOCVID2045

DISCLAIMER The *Journal of Neurosurgery* acknowledges that the following section is published verbatim as submitted by the authors and did not go through either the *Journal's* peer-review or editing process.

© 2021 The authors, CC BY 4.0 (http://creativecommons.org/licenses/by/4.0/)

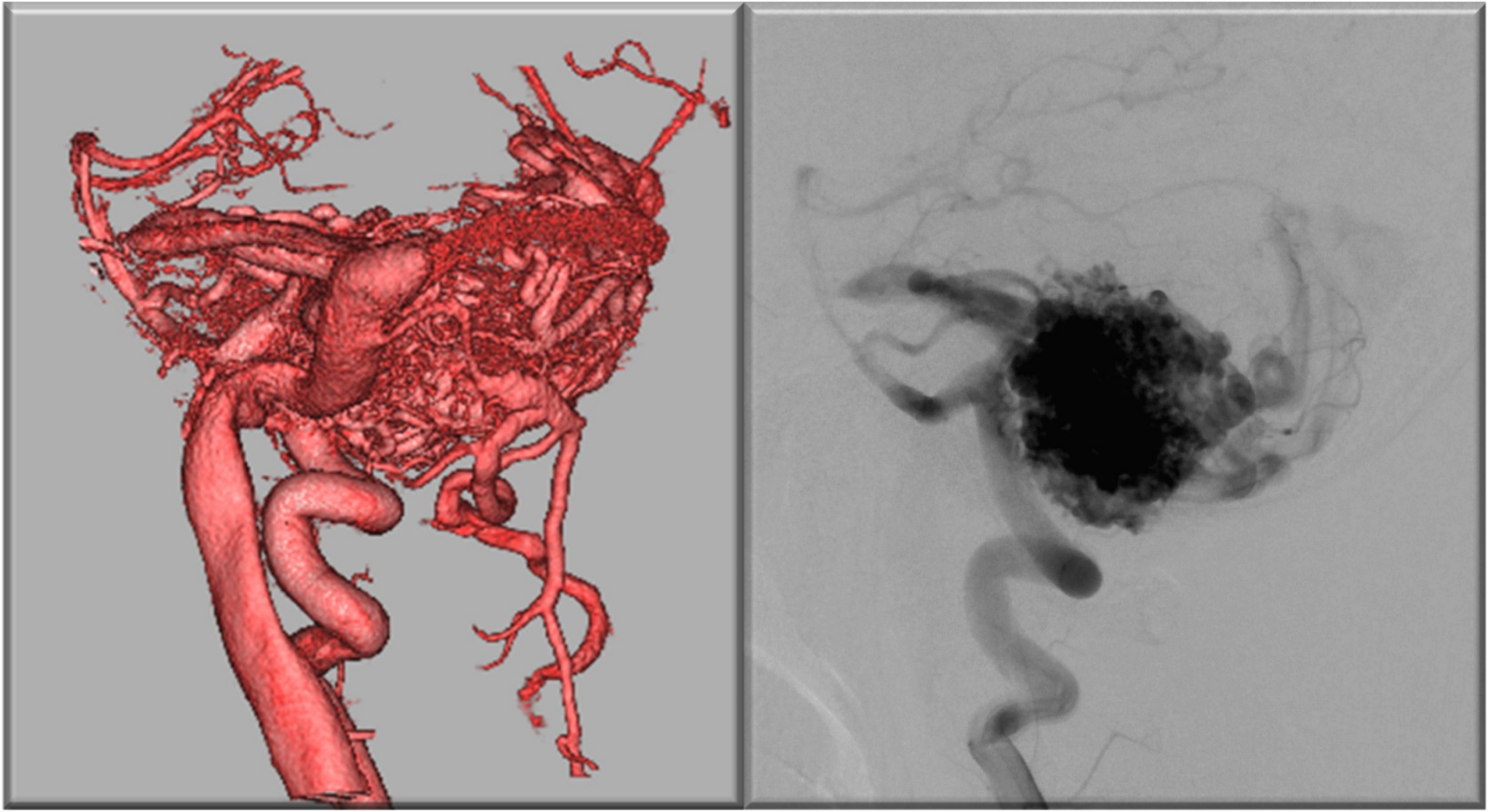

Supplemental Figure 1: The 3D-digital subtraction angiography demonstrated that the nidus is fed from left anterior inferior cerebellar artery (AICA), left posterior inferior cerebellar artery (PICA) and several feeders around the occipital sinus. The deep draining veins enter the bilateral superior petrosal sinus (SPS) and the superficial draining veins enter the occipital sinus.
